# Acute generalized exanthematous pustulosis (AGEP) in Singapore

**DOI:** 10.1186/2045-7022-4-S3-P90

**Published:** 2014-07-18

**Authors:** Haur Yueh Lee, T Thirumoorthy, Shiu Ming Pang

**Affiliations:** 1 Singapore General Hospital, Singapore; 2 Singapore General Hospital, Dermatology, Singapore

## Background

AGEP is a cutaneous adverse drug reaction characterized by acute onset of extensive sterile postulation. The incidence is estimated to be 1-5 cases and is thought to be a self-limiting reaction. The epidemiology of AGEP in Asia has not been well characterized. The aim of our study is to evaluate the epidemiology, causative drugs and clinical course of AGEP in an Asian population.

## Method

All cases of AGEP that were admitted in the Singapore General Hospital from the period of 2005-2012 were included in this study. Demographics, clinical presentation, laboratory data and outcome were retrospectively evaluated

## Results

There were 20 cases of AGEP that were seen during the study period. Of which there are 8 males (40%) and 12 (females (60%) with a mean age of 60 years (Range : 24-85 years). In addition to extensive pinpoint pustules, other cutaneous features included facial edema, target-like lesions with rim of pustules and edema bulla. Common laboratory derangement included leukocytosis, eosinophilia, atypical lymphocytes and elevated C reactive protein. 25% and 30% of cases had liver or renal impairment respectively. The most common causative drugs were betalactam antibiotics, fluoroquinolones and anti-epileptic medications. The median latency from drug initiation to onset of rash was 5 days. 90% of cases resolved within 2 days. In-hospital mortality was 10%.

## Conclusion

The epidemiology of AGEP and drug causality in Singapore corresponds to other reported series. Although AGEP is generally thought to be benign, there were a significant proportion of patients who had systemic involvement of the liver and kidney. The extent and factors for systemic complications of AGEP would need to be further defined.

